# Volatile composition, antidiabetic, and anti-obesity potential of *Brassica incana* leaf and flowering top extracts

**DOI:** 10.1080/13880209.2022.2128825

**Published:** 2022-10-11

**Authors:** Maria Fernanda Taviano, Sonia Núñez, Adrián Millán-Laleona, Concetta Condurso, Antonella Verzera, Maria Merlino, Monica Ragusa, Natalizia Miceli, Víctor López

**Affiliations:** a Department of Chemical, Biological, Pharmaceutical and Environmental Sciences, University of Messina, Messina, Italy; b Department of Pharmacy, Faculty of Health Sciences, Universidad San Jorge, Villanueva de Gállego (Zaragoza), Spain; c Instituto Agroalimentario de Aragón-IA2, CITA-Universidad de Zaragoza, Zaragoza, Spain; d Department of Veterinary Sciences, Viale Palatucci, University of Messina, Messina, Italy; e IRCCS Istituto Ortopedico Rizzoli, Complex Structure of Surgical Sciences and Technologies, Bologna, Italy

**Keywords:** Anti-glucosidase, anti-lipase, antioxidant, Brassicaceae, enzyme inhibitor, isothiocyanates, volatile compounds

## Abstract

**Context:**

*Brassica incana* Ten. (Brassicaceae) is an edible plant with very limited available information. Previous studies have demonstrated the polyphenolic profile and the antioxidant and cytotoxic properties of the leaf and flowering top hydroalcoholic extracts.

**Objective:**

The volatile composition and the antidiabetic and anti-obesity potential of *B. incana* leaf and flowering top extracts have been investigated.

**Material and methods:**

The volatile characterization of the extracts was attained by HS-SPME-GC/MS analysis. The antidiabetic and anti-obesity potential was investigated spectrophotometrically *in vitro* by the ability to modulate pancreatic lipase and α-glucosidase at different concentrations using orlistat and acarbose as reference drugs. The inhibition of advanced glycation end-products (AGEs) was measured with aminoguanidine as reference and the antioxidant activity with the xanthine/xanthine oxidase system and Trolox for comparative purposes.

**Results:**

Several volatiles belonging to different chemical classes were identified, being sulphur compounds the most abundant in both leaf and flowering top extracts (56.33% and 64.40% of all volatiles). Although the leaf extract showed lower IC_50_ values in most of the assays (0.968 and 1.921 mg/mL for α-glucosidase; 0.192 and 0.262 mg/mL for AGEs; 0.022 and 0.038 mg/mL for superoxide scavenging), there were no statistically significant differences between both samples. These extracts showed a similar behaviour to Trolox in the xanthine oxidase assay (IC_50_ values of 0.022 mg/mL for leaf extract; 0.038 mg/mL for flowering top and 0.028 for Trolox).

**Conclusions:**

Leaves and flowering tops from *B. incana* can be used as sources of functional compounds that could act as antidiabetic and anti-obesogenic agents.

## Introduction

*Brassica incana* Ten., a wild *B. oleracea*-related species, is an edible plant belonging to the Brassicaceae family. *Brassica incana* is a suffrutex up 100 cm high, woody at the base, branched, glabrous except at base. Basal leaves with petiole with two irregularly dentate wings, are pubescent to tomentose especially on the lower surface and along the veining, ovate to lanceolate, lyrate; the lamina has margin entire and irregularly crenate or 1–2 lobes in the lower half, usually obtuse; upper leaves are denticulate, with amplexicaule basal auricles, gradually smaller. The flowers are gathered in racemes many-flowered with yellow spatulate petals. The fruit is a siliqua patent, constricted at intervals, terete, gradually attenuate into beak (Heywood 1964). This species is native to south-eastern Europe, including Albania, Bosnia-Herzegovina, Croatia, Greece, and Italy; the plant has also been introduced in Ukraine and Crimea (Marhold 2011).

As far as we know, only a few studies on *B. incana* are present in the published literature, which focus on the glucosinolates contained in the leaves and the seeds, while no studies on its therapeutic potential are available (Horn and Vaughan 1983; Heaney et al. 1987; Velasco and Becker 2000).

Considering the very limited information about *B. incana*, our research team started a study aimed at investigating the potential of this species as a source of bioactive phytochemicals. In a previous study, some of the authors of the present work had characterized the volatile composition of *B. incana* fresh leaves and roots (Tripodi et al. 2012). In a recent work, the antioxidant properties, the cytotoxicity against human colorectal adenocarcinoma (Caco-2) cells and the absence of toxicity versus brine shrimp larvae (*Artemia salina* Leach) of the hydroalcoholic extracts obtained from the leaves and the flowering tops of *B. incana* grown wild in Sicily (Italy) were established (Miceli et al. 2020). Moreover, the quali-quantitative characterization of the phenolic compounds was performed, highlighting the presence of quercetin, kaempferol and isorhamnetin derivatives, whose antioxidant, anti-inflammatory properties as well as protective against metabolic disorders have been previously demonstrated (Carullo et al. 2017; Nasri et al. 2017; Liu et al. 2021).

As a continuation of the ongoing research, this work was designed to further investigate the phytochemical volatile profile and the biological potential of the same extracts obtained from edible parts of this species. To achieve a comprehensive view of the volatile composition of the extracts the headspace solid-phase microextraction (HS-SPME) coupled to gas chromatography-mass spectrometry (GC-MS) was utilized. Besides, *B. incana* extracts were investigated *in vitro* as candidates with antidiabetic and anti-obesity potential. For this purpose, the ability to modulate lipase and α-glucosidase together with inhibition of advanced glycation end-products (AGEs) and free radicals was examined (Sriramavaratharajan and Murugan 2018; Mustafa et al. 2022).

## Materials and methods

### Reagents and chemicals

All enzymes for bioassays such as lipase, α-glucosidase and xanthine oxidase were obtained from Sigma-Aldrich (Madrid, Spain). Orlistat as drug reference was also acquired from Sigma-Aldrich (Madrid, Spain) while acarbose was bought in Cymit Quimica (Barcelona, Spain). All other reagents, unless indicated, were purchased from Sigma (St. Louis, MO).

### Plant material and extraction procedure

The plant material was collected around Capo d’Orlando (Messina, Italy). The leaves of *Brassica incana* were harvested in November 2018 and the flowering tops in May 2019. The taxonomic identification was confirmed by Prof. S. Ragusa, Department of Health Sciences, University Magna Graecia of Catanzaro. A voucher specimen (1108/18) was deposited in the same department.

After harvesting, the plant material was washed, blended, frozen, and lyophilized. The extraction was carried out as reported in our previous work (Miceli et al. 2020). The yields of the leaf and flowering top hydroalcoholic (70% MeOH) extracts, compared to 100 g of lyophilized plant material, were 26.47% and 33.16%, respectively.

### Characterization of volatile compounds by SPME-GC/MS

#### Extraction (HS-SPME)

The hydroalcoholic extracts of both the leaves and the flowering tops of *B. incana* were analyzed for their volatile composition by HS-SPME-GC/MS.

The dried extracts were solubilized in saturated sodium chloride solution to a final concentration of 10 mg/mL; then 3 ± 0.1 mL of each extract solution were transferred to a 7 mL vial closed with a ‘mininert’ valve (Supelco, Bellefonte, PA). For the volatile extraction, the sample was equilibrated for 15 min at 40 °C, and a DVB/CAR/PDMS fibre, 50/30 μm film thickness (Supelco, Bellefonte, PA), was exposed for 15 min to the headspace of the sample maintained at 40 °C under continuous magnetic stirring. Finally, the SPME fibre was placed for 3 min into the injector port of the GC/MS, held at 260 °C, for the thermal desorption of the analytes onto the capillary GC column.

#### Analysis (GC/MS)

The volatiles were analyzed by a Shimadzu GC 2010 Plus gas chromatograph coupled to a TQMS 8040 triple quadrupole mass spectrometer (Shimadzu, Milan, Italy). Two capillary columns of different polarity were used: (1) VF-WAXms, 60 m, 0.25 mm i.d., 0.25 μm film thickness polar column (Agilent Technologies Italia S.p.A., Milan, Italy); (2) DB-5ms, 30 m, 0.25 mm i.d., 0.25 μm film thickness apolar column (Agilent Technologies Italia S.p.A., Milan, Italy).

The conditions were as follows. Injection mode: splitless. Oven temperature: (1) 45 °C held for 5 min, then increased to 80 °C at a rate of 10 °C/min and to 240 °C at 2 °C/min, held at 240 °C for 5 min, for VF-WAXms column; (2) 45 °C increased to 160 °C at a rate of 3 °C/min and to 260 °C at 10 °C/min, held at 260 °C for 5 min, for DB-5ms column. Carrier gas: helium at a constant flow of 1 mL/min. Transfer line temperature: 250 °C. Acquisition range: 40–360 *m*/*z*; scan speed of 1250. For the identification of the volatiles, mass spectral data, NIST’ 14 (NIST/EPA/NIH Mass Spectra Library, version 2.0, USA) and FFNSC 3.0 database, linear retention indices (LRI), literature data and injection of the available standards were used (Cincotta et al. 2018).

### Bioactivity of B. incana extracts

All *in vitro* bioactivity tests were performed as previously described in Taviano et al. (2020) using control wells with all reagents except for extract and sample wells in order to check the inhibitory profile. Blank wells were also measured in order to eliminate interferences. A wide range of concentrations (0.0001–10 mg/mL for enzymatic assays and 0.03–0.5 mg/mL for AGES) was tested in the assays.

#### Pancreatic lipase inhibition

Lipase inhibition was measured as previously reported by Taviano et al. (2020) in 96-well microplates. Briefly, 40 µL of enzyme (2.5 mg/mL in 0.1 M phosphate buffer, pH 7.0), previously centrifugated at 2000 × *g* for 7 min was mixed with 40 µL of extract and 20 µL of 10 mM *p*-nitrophenyl butyrate (*p*-NPB). After 10 min incubation, absorbance was recorded at 405 nm using also orlistat as drug reference.

#### α-Glucosidase inhibition

α-Glucosidase inhibition was also investigated as reported by Taviano et al. (2020). 100 µL of enzyme (1 U/mL) dissolved in buffer (12.5 mM Na_2_HPO_4_, 3.3 mM NaH_2_PO_4_; pH = 6.9) was mixed with 50 µL of extract and then incubated at room temperature for 10 min. Then, 50 µL of 3 mM *p*-nitrophenyl-α-d-glucopyranoside (pNPG) were added. After 15 min at 37 °C, absorbance was recorded at 405 nm using acarbose as drug reference.

#### Advanced glycation end-products inhibition

Advanced glycation end-products (AGEs) inhibition was measured in 96-black well-plates according to Spínola and Castilho (2017). 10 mg/mL Bovine serum albumin solution (50 μL) was mixed with 80 μL of 0.1 M phosphate buffer (containing sodium azide 3 mM, pH =7.4), 50 μL of 0.5 M fructose solution (0.5 M) and 20 μL of extracts. After 24 h incubation at 37 °C in the dark, fluorescence was measured (355 nm excitation wavelength and 460 nm emission wavelength) using aminoguanidine (AMG) as drug reference.

#### Antiradical activity

Free radical scavenging activity was evaluated by calculating the percentage of inhibition of superoxide radicals generated by xanthine oxidase (Mustafa et al. 2022) using trolox as reference substance. Briefly, 240 μL of the reaction mixture [90 μM xanthine, 16 mM Na_2_CO_3_, and 22.8 μM nitroblue tetrazolium chloride (NBT) in phosphate buffer pH 7.0] was mixed with 30 μL of extract solution at different concentrations; then, xanthine oxidase (XO) was added, and absorbance was read at 560 nm after 2 min incubation at 37 °C.

### Data and statistical analyses

Results about bioactivity are presented as mean values and standard error of mean (SEM) of at least three independent experiments in different days. All bioactivity assays were performed at between 5 and 9 different concentrations for non-linear regression. GraphPad Prism v.7.0 (GraphPad Software, La Jolla, CA) was used for formal analyses. IC_50_ values were obtained by non-linear regression and one-way ANOVA with Tukey multiple comparison test was used in order to detect differences between the samples.

## Results and discussion

### Characterization of volatile compounds by SPME-GC/MS

The volatile composition of the hydroalcoholic extracts of the *B. incana* leaves and flowering tops are reported in Tables 1 and 2, respectively. A large number of compounds belonging to the chemical classes of esters, alcohols, acids, ketones, aldehydes, terpenes, hydrocarbons, sulphur compounds and nitriles were detected in the headspace of leaf and flowering top extracts.

**Table 1.** Composition as volatile constituents and classes of substances of *B. incana* leaf hydroalcoholic extract.

Compound	LRI^a^ on DB-5ms	LRI^a^ on VF-WAXms	Amount^b^	Percentage
Sulphur compounds				
Dimethyl disulphide	742	1080	3959	1.99
Isobutyl isothiocyanate	929	1322	610	0.31
Dimethyl trisulphide	969	1388	21,163	10.66
3-Butenyl isothiocyanate	973	1462	85,532	43.10
Methyl methylthiomethyl disulphide	1135	1665	520	0.26
All			111,784	56.33
Nitriles				
4-Pentenenitrile	745	1279	5254	2.65
Heptanenitrile	991	1408	120	0.06
All			5374	2.71
Aldehydes				
(*E*)-2-Pentenal	758	1131	387	0.19
Heptanal	904	1186	776	0.39
(*E*)-2-Heptenal	958	1329	571	0.29
Octanal	1004	1290	1116	0.56
(*E*)-2-Octenal	1058	1433	140	0.07
Nonanal	1105	1396	4201	2.12
Decanal	1207	1501	7448	3.75
Undecanal	1307	1606	240	0.12
Dodecanal	1408	1711	1174	0.59
Tridecanal	1510	1817	559	0.28
Tetradecanal	1610	1922	1058	0.53
β-Cyclocitral	1220	1624	1388	0.70
Safranal	1200	1649	1071	0.54
All			20,128	10.14
Ketones				
6-Methyl-5-hepten-2-one	985	1340	249	0.13
All			249	0.13
Alcohols				
1-Hexanol	870	1347	188	0.09
1-Octen-3-ol	980	1448	233	0.12
2-Ethyl-1-hexanol	1028	1489	484	0.24
(*Z*)-2-Octen-1-ol	1060	1620	356	0.18
(*E*)-2-Octen-1-ol	1068	1616	101	0.05
1-Octanol	1071	1557	171	0.09
1-Nonanol	1173	1659	363	0.18
Dodecanol	1475	1966	15,106	7.61
Tetradecanol	1576	2171	5164	2.6
All			22,165	11.17
Acids				
Butanoic acid	779	1636	5276	2.66
Hexanoic acid	978	1851	7856	3.96
Octanoic acid	1171	2064	3707	1.87
Hexadecanoic acid	1960	3000	2426	1.22
All			19,264	9.71
Esters				
3-Methyl-1-butyl acetate	876	1121	829	0.42
Hexyl acetate	1011	1270	3001	1.51
1-Methylbutyl butanoate	1015	1208	509	0.26
Butyl hexanoate	1185	1412	664	0.33
Hexyl butanoate	1194	1415	228	0.11
Methyl hexadecanoate	1926	2216	3268	1.65
All			8498	4.28
Terpenes				
Limonene	1029	1193	4363	2.20
(*Z*)-Calamenene	1531	1835	1335	0.67
Guaiol	1597	2087	2333	1.18
Bulnesol	1667	2208	2391	1.20
All			10,423	5.25

^a^Linear retention indexes calculated according to the Van Den Dool and Kratz equation.

^b^Peak area arbitrary scale.

**Table 2.** Composition as volatile constituents and classes of substances of *B. incana* flowering top hydroalcoholic extract.

Compounds	LRI^a^ on DB-5ms	LRI^a^ on VF-WAXms	Amount^b^	Percentage
Sulphur compounds				
Dimethyl disulphide	742	1080	32,307	18.96
Isobutyl isothiocyanate	929	1322	137	0.08
Dimethyl trisulphide	969	1388	63,208	36.62
3-Butenyl isothiocyanate	973	1462	995	0.57
Dimethyl tetrasulfide	1216	1750	14,244	8.16
All			112,673	64.40
Nitriles				
3-Methyl-3-butenenitrile	759	–	28,026	16.06
Benzyl nitrile	1136	1893	1442	0.83
Benzenepropanenitrile	1237	2041	119	0.07
1H-Indole-3-acetonitrile	1807	–	1028	0.59
All			30,495	17.54
Aldehydes				
Hexanal	803	1085	683	0.39
Heptanal	904	1186	110	0.06
(*E*)-2-Heptenal	958	1329	299	0.17
Benzaldehyde	962	1530	231	0.13
(*E.E*)-2.4-Heptadienal	1000	1508	92	0.05
Octanal	1004	1290	400	0.23
Phenylacetaldehyde	1044	1645	336	0.19
Nonanal	1105	1396	1150	0.66
Decanal	1207	1501	356	0.20
All			3657	2.10
Ketones				
1-Penten-3-one	721	1020	3405	1.95
6-Methyl-5-hepten-2-one	985	1340	251	0.14
6-Methyl-3.5-heptadien-2-one	1096	1582	532	0.30
6.4.10-Trimethyl-2-pentadecanone (hexahydrofarnesyl acetone)	1844	2119	2302	1.32
All			6490	3.72
Alcohols				
1-Octen-3-ol	980	1448	1871	1.07
(*E*)- 2-Octen-1-ol	1068	1616	867	0.50
1-Octanol	1071	1557	1945	1.11
1-Nonanol	1173	1659	98	0.06
All			4781	2.74
Acids				
3-Methylbutanoic acid	834	1681	417	0.24
2-Methylbutanoic acid	845	1687	874	0.50
(*Z*)-3-Hexenoic acid	992	1940	80	0.05
2-Methyl-4-pentenoic acid	996	–	315	0.18
2-Ethylhexanoic acid	1115	1129	165	0.09
Octanoic acid	1171	2064	665	0.38
Nonanoic acid	1268	2165	836	0.48
Decanoic acid	1365	2267	386	0.22
All			3738	2.14
Esters				
3-Methyl-1-butyl acetate	876	1121	290	0.17
Pentyl 2-methylpropanoate	1056	1243	254	0.15
Phenyl acetate	1064	1660	86	0.05
Methyl octanoate	1127	1411	1162	0.67
Carveyl acetate	1314	2071	150	0.09
Octyl 2-methylpropanoate	1347	1547	89	0.05
Benzyl 3-methylbutanoate	1392	1852	121	0.07
1-Methylethyl decanoate	1428	1615	409	0.23
1-Octen-3-yl hexanoate	1507	–	328	0.19
1-Methylethyl tetradecanoate	1826	2017	57	0.03
Methyl hexadecanoate	1926	2216	1563	0.90
Methyl linoleate	2095	2480	76	0.04
Methyl linolenate	2101	2503	357	0.20
All			4790	2.83
Terpenes				
α-Pinene	933	1025	149	0.09
β-Pinene	978	1108	333	0.19
p-Cymene	1025	1270	600	0.34
Limonene	1029	1193	3632	2.20
Eucalyptol	1033	1206	715	0.41
α-Isophorone	1124	1621	805	0.46
Safranal	1200	1639	451	0.26
β-Cyclocitral	1220	1623	530	0.30
Citronellol	1232	1757	68	0.04
Carvacrol	1299	2225	110	0.06
Orivone	1354	–	133	0.08
All			7740	4.43
Hydrocarbons				
Heptadecane	1700	1700	59	0.03
Octadecane	1800	1800	40	0.02
1-Eicosene	1994	2051	69	0.04
All			168	0.10

*Linear retention indexes calculated according to the Van Den Dool and Kratz equation.

^b^Peak area arbitrary scale.

 Regarding the leaf extract, its volatile fraction was constituted mainly of sulphur compounds (sulphides and isothiocyanates) which accounted for over 56% of all volatiles. Among the other chemical classes, alcohols, aldehydes and acids were the most represented with a percentage close to 10% for each one. 3-Butenyl isothiocyanate (43.10%), dimethyl trisulphide (10.66%) and 1-dodecanol (7.61%) were the compounds quantitatively most represented.

These results are quite different from those reported in our previous study on the volatiles of *B. incana* leaves (Tripodi et al. 2012); this can be explained considering that previously the SPME extraction technique was directly applied to the fresh plant leaves, and the characteristic “green leaf” volatiles, such as (*E*)-2-hexanal (leaf aldehyde), (*Z*)-3-hexenol (leaf alcohol) and, in general, C6 aldehydes and alcohols, resulted the main constituents of the leaf headspace; instead isothiocyanates were the main volatiles of the hydroalcoholic extract of *B. incana* leaves. Isothiocyanates arise from the glucosinolate hydrolysis after plant cell rupture, and in case of the hydroalcoholic extract, the procedure for sample preparation certainly favoured their formation (Fenwick et al. 1983). However, in both cases the class of isothiocyanates was constituted mostly of 3-butenyl isothiocyanate.

The volatile fraction of the flowering top extract was composed mostly of sulphur compounds and nitriles. These two classes of compounds constituted about the 82% of the whole volatile fraction. The main constituents were dimethyl trisulphide (36.22%), dimethyl disulphide (18.51%), 3-methyl-3-butenenitrile (16.06%) and dimethyl tetrasulfide (8.16%). Isobutyl isothiocyanate and 3-butenyl isothiocyanate were the only isothiocyanates detected but they were present at very low levels representing only the 0.08% and 0.57% of the whole volatile fraction, respectively. The remaining compounds were present as minor constituents (<1%), except for 1-penten-3-one (1.95%), hexahydrofarnesyl acetone (1.32%), 1-octen-3-ol (1.07%), 1-octanol (1.11%) and limonene (2.20%).

The volatile profiles of the *B. incana* extracts showed significant differences. In particular, among sulphur compounds, isothiocyanates prevailed in the leaf extract, whereas sulphides in the flowering top one; similarly, Robertson et al. (1993) analyzing five different varieties of *Brassica napus* found that organic sulphides were among the major volatile compounds released from the flowers, whereas no isothiocyanates were detected.

Moreover, the headspace of the flowering top extract was very rich in nitriles while aldehydes, alcohols and acids were quantitatively less represented than in the leaf extract headspace.

Like isothiocyanates, nitriles are hydrolysis products of glucosinolates by the action of the myrosinase. The enzymatic cleavage can lead to different products depending on the glucosinolate structure and the presence of factors which modify the action of the enzyme. It has been demonstrated that ferrous ions and acidic conditions favour nitrile formation; moreover, nitriles are also favoured by the aglycone autolysis (Fenwick et al. 1983).

### Bioactivity of B. incana extracts

**Figure 1.**
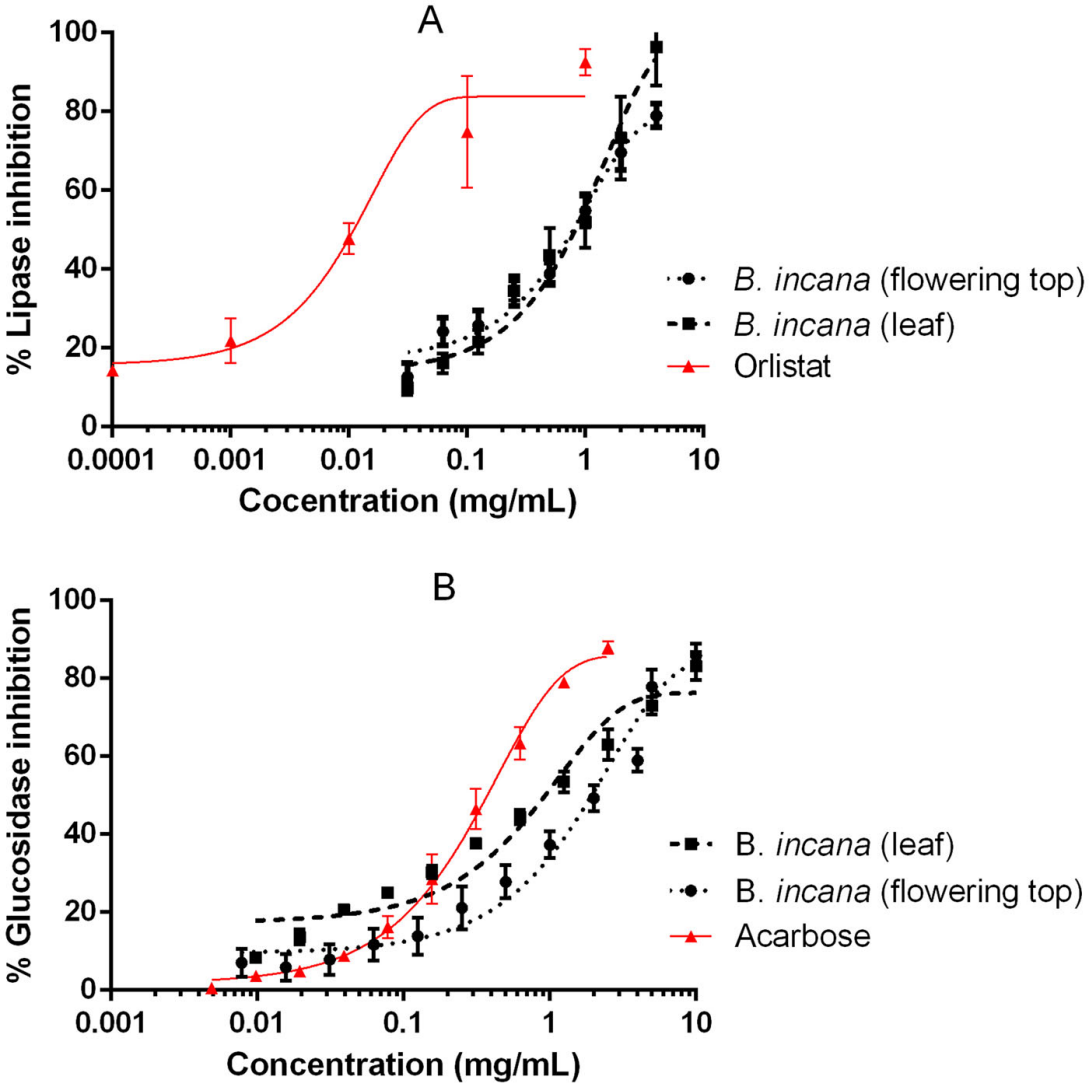
Inhibition of pancreatic lipase (A) and α-glucosidase (B) by *B. incana* leaf and flowering top hydroalcoholic extracts. Orlistat and acarbose were used as positive control substances.

*Brassica incana* extracts were able to inhibit pancreatic lipase and α-glucosidase in a dose-dependent manner, as reported in Figure 1. Although the observed inhibitions were not superior to the drug references used in the bioassays, orlistat and acarbose, it is the first time that these activities are reported for this plant species. In the case of pancreatic lipase, the IC_50_ value was lower for the flowering top extract whereas in the glucosidase assay the best results were obtained for the leaves extract (Table 3). Both enzymes, pancreatic lipase and α-glucosidase, are key physiological and pharmacological targets for the treatment and prevention of metabolic disorders such as obesity, diabetes or the metabolic syndrome and therefore widely studied as pharmacological targets for phytochemicals (Ahmad et al. 2020; El-Nashar et al. 2021). Cruciferous plants (Brassicaceae family) have been presented several times as healthy food plants due to their content in bioactive compounds; particularly because they are rich in glucosinolates and their derived volatile sulphur compounds known as isothiocyanates, which are in relation with the prevention of certain cancers and disorders such as the metabolic syndrome (Esteve 2020; Melim et al. 2022). Although is it not clear in the literature that isothiocyanates act as pancreatic lipase or alpha-glucosidase inhibitors, other cruciferous plants have also demonstrated this kind of *in vitro* activity (Taviano et al. 2020). In previous work, several polyphenols, such as phenolic acids or flavonoids, have been detected in both *B. incana* leaf and flowering top extracts utilized in this study (Miceli et al. 2020). It was reported that extracts rich in polyphenols have a great capacity to inhibit enzymes involved in glucose and fat metabolism, such as alpha-glucosidase and lipase (Les et al. 2020). Thus, it can be assumed that the polyphenolic compounds are involved in the lipase or glucosidase inhibiting activity highlighted for *B. incana* extracts.

**Table 3.** IC_50_ values for *B. incana* leaf and flowering top hydroalcoholic extracts and drug compounds used as references.

Samples	IC_50_ values (mg/mL) in different bioassays			
	AGEs	Superoxide	Glucosidase	Lipase
*B. incana* (leaf)	0.192 ± 0.024^a^	0.022 ± 0.003^a^	0.968 ± 0.141^a^	1.086 ± 0.319^a^
*B. incana* (flowering top)	0.262 ± 0.020^a^	0.038 ± 0.012^a^	1.921 ± 0.321^a^	0.939 ± 0.131^a^
AMG	0.0744 ± 0.017^b^	–	–	–
Trolox	–	0.028 ± 0.001^a^	–	–
Acarbose	–	–	0.306 ± 0.039^b^	–
Orlistat	–	–	–	0.0277 ± 0.015^b^

Results are expressed as average ± SEM of at least three independent experiments. ^a,b^ Different letters within the same column indicate significant differences between mean values (*P* < 0.05). No significant differences were found between leaves and flowering tops using ANOVA and Tukey for multiple comparison statistical analyses.

**Figure 2.**
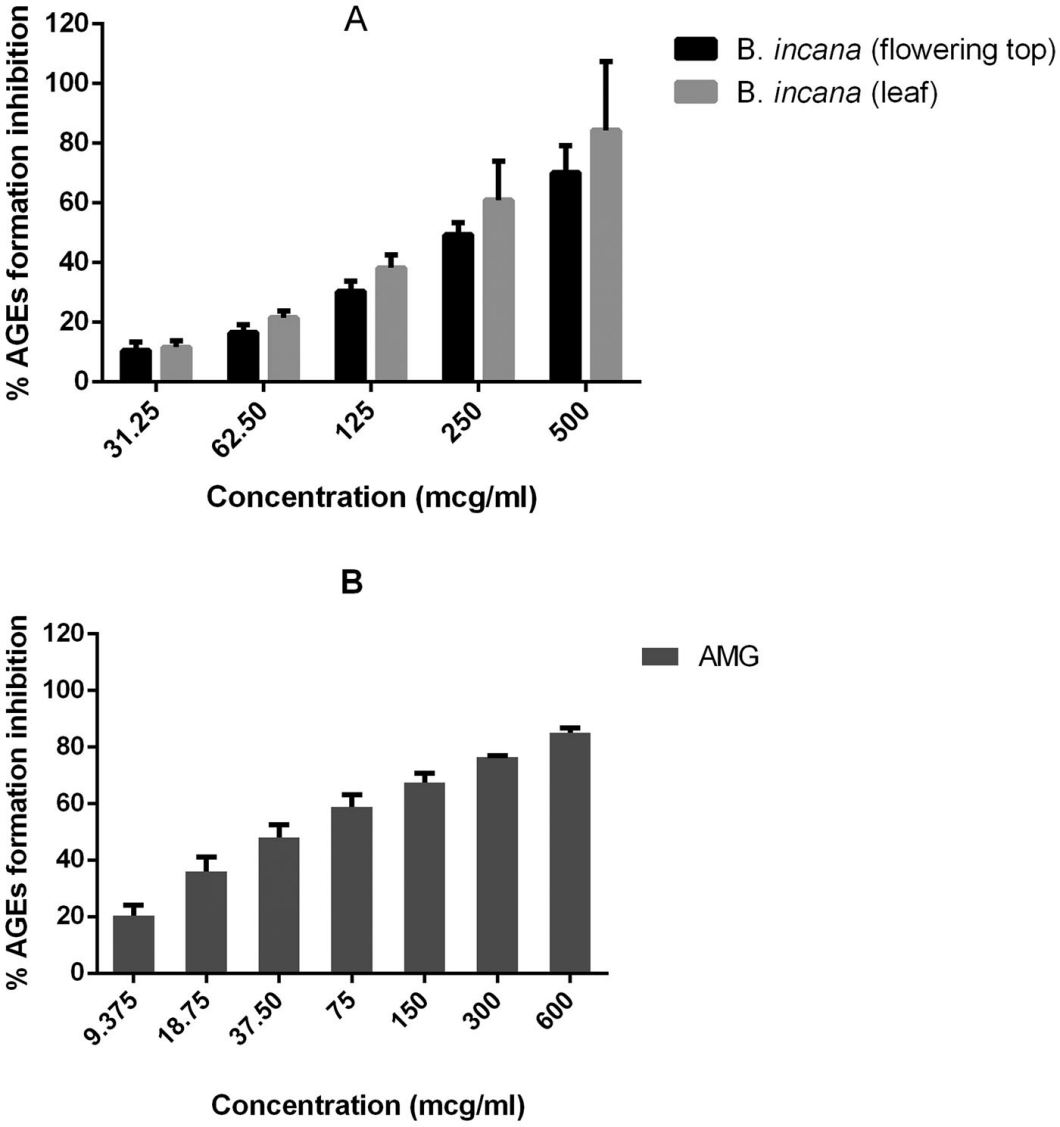
Inhibition of advanced glycation end-products (AGEs) by *B. incana* leaf and flowering top hydroalcoholic extracts (A) compared to aminoguanidine (B), used as positive control substance.

In relation with antiobesogenic and antidiabetic activity, these extracts have also revealed AGEs inhibitory properties. The inhibitory activity is also better for the leaves than for the flowering top extracts (Figure 2); the level of fluorescence of BSA alone, and BSA + fructose, and BSA + fructose + treatments is included as Supplementary material. AGEs production is implicated in these metabolic diseases because of bad hyperglycaemia control; elevated glucose blood concentration leads to increased protein glycation generating a proinflammatory state (Garay-Sevilla et al. 2021).

**Figure 3.**
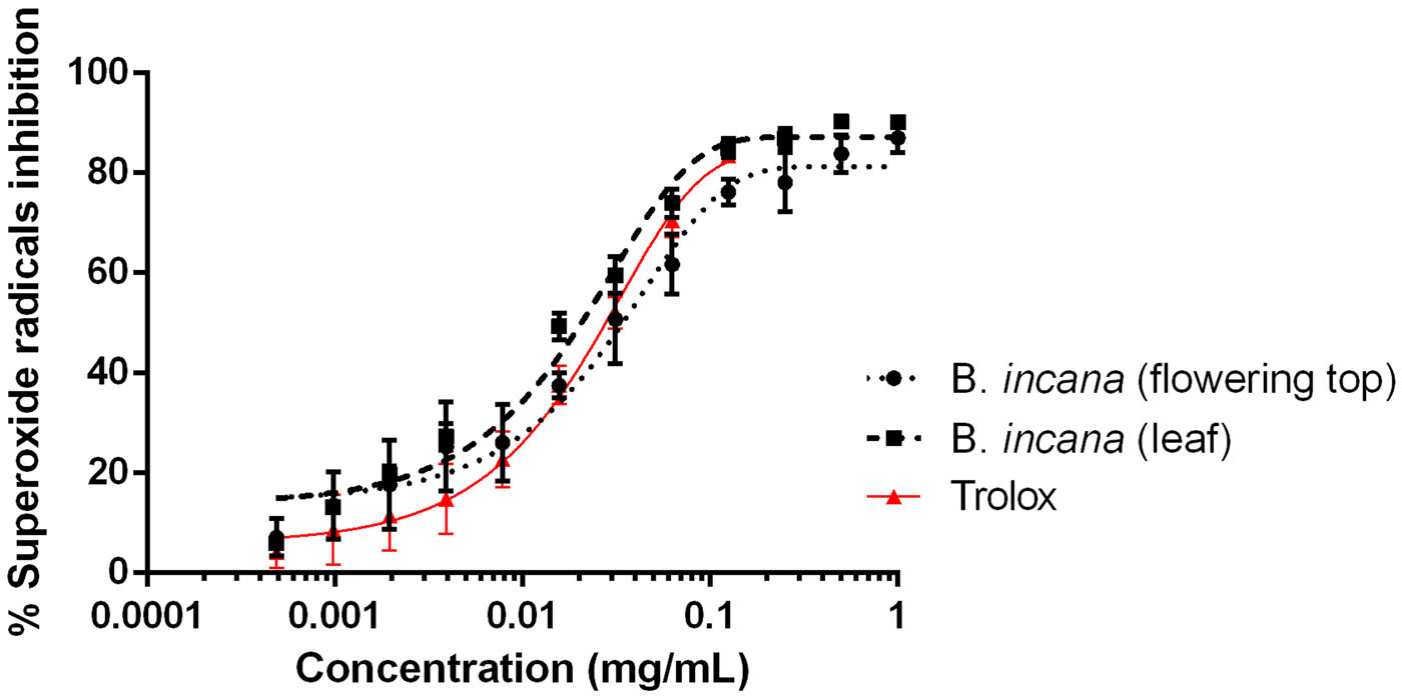
Antioxidant activity against superoxide radicals by *B. incana* leaf and flowering top hydroalcoholic extracts. Trolox was used as positive control substance.

Nevertheless, hyperglycaemia not only contributes to AGEs production but also to oxidative stress and free radical release, inducing cellular ageing and disfunction (Silveira Rossi et al. 2022). For this reason, it is also important that α-glucosidase inhibitors may also act as anti-AGEs and antioxidant agents. Figure 3 shows the activity of leaf and flowering top extracts of *B. incana* against superoxide radicals in the xanthine/xanthine oxidase system. As it can be observed, the antiradical activity is slightly better for the leaf extract, whose IC_50_ values are lower than the values obtained for the flowering top one (Table 3). The antioxidant activity of *B. incana* has already been published (Miceli et al. 2020; Picchi et al. 2020) but this is the first time that is performed against superoxide radicals generated by xanthine oxidase and compared with trolox using non-linear regression analysis. Our results are in accordance with the previous work made by the authors as the leaf extract is better as radical scavenger than the flowering top (Miceli et al. 2020); nevertheless, it is surprising that the capacity of our extracts to inhibit superoxide radicals is even better than the activity displayed by Trolox (Figure 3 and Table 3). Previous work, as by Miceli et al. (2020), have also dealt with the presence of phenolics and have demonstrated the absence of toxicity against *A. salina* nauplii, which is also important to recommend a plant matrix as a healthy functional food. Considering that these extracts act as enzyme inhibitors of pancreatic lipase and α-glucosidase and as antioxidant and anti-AGEs agents, they could represent an interesting source of bioactive molecules.

## Conclusions

Herein, the volatile profile and the antidiabetic and anti-obesity potential of leaves and flowering tops from *Brassica incana* grown wild in Sicily (Italy) are reported. Significant differences in the volatile composition of the leaf and flowering top hydroalcoholic extracts have been highlighted. In particular, among sulphur compounds, isothiocyanates prevailed in the former, being 3-butenyl isothiocyanate the main component. Both extracts have been shown for the first time to inhibit pancreatic lipase, α-glucosidase, advanced glycation end-products and superoxide radicals in the xanthine/xanthine oxidase system, although the flowering top extract displayed better pancreatic lipase inhibiting activity, there were not significant differences between the leaf extract and the flowering top. The present findings indicate that leaves and flowering tops from *B. incana* are a source of functional ingredients that could act as antidiabetic and anti-obesogenic agents.

## Supplementary Material

Supplemental Material
