# Supplementary material for: Volatile composition, antidiabetic, and anti-obesity potential of Brassica incana leaf and flowering top extracts
Source: Pharm Biol. 2022 Oct 11;60(1):1994–2001. doi: 10.1080/13880209.2022.2128825 (PMC9559316; doi:10.1080/13880209.2022.2128825)
Supplement: Supplemental Material [file IPHB_A_2128825_SM1644.pdf]

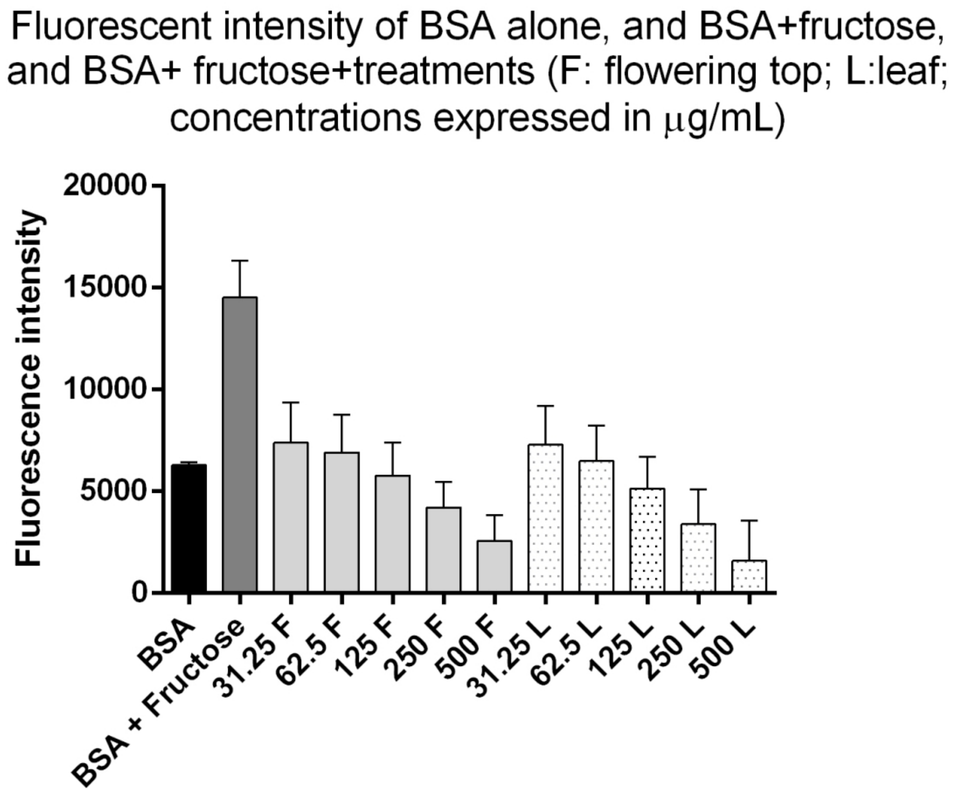

124x106mm (300 x 300 DPI)
